# The impact of stimulation rates in vestibular evoked myogenic potential testing

**DOI:** 10.5935/1808-8694.20130106

**Published:** 2015-10-08

**Authors:** Aline Tenório Lins Carnaúba, Otávio Gomes Lins, Ilka do Amaral Soares, Kelly Cristina Lira de Andrade, Pedro de Lemos Menezes

**Affiliations:** a MSc. Student, Human Communication Health, Federal University of Pernambuco (Speech and Hearing Therapist).; b PhD, Medicine (Neurology), UNIFESP (Adjunct Professor, Federal University of Pernambuco).; c MSc., Human Communication Disorders, UNIFESP (Assistant Professor, Alagoas State University of Health Sciences - UNCISAL).; d PhD, Physics Applied to Medicine, USP (Professor, Alagoas State University of Health Sciences - UNCISAL - UNCISAL). Federal University of Pernambuco.

**Keywords:** acoustic stimulation, vestibular evoked myogenic potential, vestibular function tests

## Abstract

Vestibular evoked myogenic potentials (VEMP) have been used in complementary otoneurological assessment, but the use of VEMP in clinical settings is limited. VEMPs can be used to assess vestibular function, particularly of the saccule, the inferior vestibular nerve, and/or the vestibular nucleus.

**Objective:**

To verify the highest possible - and reliable - stimulation rate to obtain VEMPs.

**Method:**

The VEMPs of 18 subjects were acquired using stimulation rates ranging between 5.1 and 40.8 stimuli per second. Study design: cross-sectional contemporary cohort study.

**Results:**

Latencies were kept unaltered and amplitudes were progressively reduced as stimulation rates were increased. However, ANOVA and the Kruskal-Wallis test failed to find statistically significant differences between the tested parameters. The study further indicated that when stimulation rates of 5.1 and 10.2 stimuli per second were compared, no statistically significant differences were observed in latency.

**Conclusion:**

The highest reliable stimulation rate observed in the group of young adults with normal hearing included in this study was 10.2 stimuli per second.

## INTRODUCTION

Vestibular evoked myogenic potentials (VEMP) have been used in the complementary assessment of vestibular function, particularly of the saccule, the inferior vestibular nerve, and/or the vestibular nucleus^1^, ^2^, ^3^, ^4^, ^5^, ^6^.

Responses are captured from neck muscles through surface electrodes. The tracings derived from acoustic stimulation are made up of two complexes of two-phase waves: p13 and n23^1^, ^7^, ^8^, ^9^, ^10^, ^11^. VEMPs can be obtained from air, bone, and galvanic acoustic stimulation^4^.

Response characteristics are correlated to the type of stimulation and frequencies used. Tone bursts^11^, ^12^, ^13^, ^14^, ^15^ or clicks^16^, ^17^ can be used in acoustic stimulation. Lower frequencies produce more homogeneous responses, with 500 Hz^6^, ^12^, ^18^, ^19^, ^20^ as the most effective frequency. Response is analyzed by the selection of peaks and assessment of amplitudes and latencies^21^, ^22^.

In the clinical setting, VEMP testing presents a series of favorable traits, as it is an objective, non-invasive, easy-to-perform test that does not bring discomfort to patients^3^, ^14^. However, there is no agreed standard to obtain VEMPs, and a wide array of methods and protocols have been used^23^. The most frequently described stimulation rate is 5 Hz. Nonetheless, higher rates, if reliable, would expedite the testing protocol.

This study aims to find the highest reliable stimulation rate to obtain VEMPs.

## METHOD

This study was approved by the institution's Research Ethics Committee and given permit 990/09. All participants signed informed consent terms before joining the study.

Eighteen individuals (36 ears) were enrolled in the study as per the following criteria: ages between 18 and 35 years and auditory thresholds equal to or lower than 20 dBNA with differences between ears per frequency of 10 dB or under. The number of participants was calculated based on the sample size for an infinite population with an alpha of 0.05, a standard deviation of 9 µV and tolerable error of 3 µV.

Individuals exposed to occupational or leisure noise, previously submitted to middle or inner ear surgery, with more than three cases of outer or middle ear infection in the current year, prior use of ototoxic medication, presence of systemic alterations conducive to vestibulocochlear involvement such as diabetes, high blood pressure, hormonal disorders, and presence of tinnitus, vertigo, dizziness or other vestibulocochlear alterations were excluded.

Participants were asked to answer a questionnaire for screening purposes. The following procedures were then carried out: otoscopic examination, pure-tone audiometry, and VEMP testing.

Surface electrodes positioned on the subjects' skin were used to record VEMPs. The positive electrode was placed on the middle third of the sternocleidomastoid (SCM) muscle on the side where stimulation was applied; the negative electrode was positioned at the level of the tendon of the SCM, just above the clavicle; and the ground electrode was placed on the frontal middle line. Patients were seated during the acquisition of SCM records, with their heads in maximum lateral rotation turned to the opposite side of stimuli application.

In VEMP examination, the mean value for 200 tone burst stimuli at 500 Hz was calculated, with stimulation rates set at 5.1, 10.2, 20.4, and 40.8 stimuli per second at an intensity of 95 dBNAn with subjects wearing ER-A3 ear buds. A pass-band filter (5-1000 Hz) with exhibition of 10 to 25 µV per division was used. Stimulation rates were not set as integer numbers in order to prevent potentials from being acquired in phase with the frequency of the Brazilian grid, set at 60 Hz^24^, ^25^. Records were captured in 40 ms windows to encompass all responses^14^, ^15^.

Wave morphology was used in the interpretation of test findings. Waves p13 and n23 were delimited by the latencies of the first positive and negative peaks by two authors/examiners. Discrepancies between authors/examiners were resolved by a third author/examiner.

### Statistical method

The data sets were treated and processed using application Predictive Analytics SoftWare (PASW^®^ Statistic) release 17.0. Mean values were presented in tables and graphs, along with standard deviations and percentile distributions.

Latency and amplitude normality for waves p13 and n23 was analyzed through the Kolmogorov-Smirnov test. ANOVA was used to compare latencies and amplitudes at different stimulation rates (5.1, 10.2, 20.4, and 40.8 stimuli per second) obtained in VEMP testing, and pairs were compared using the Tukey or the Kruskal-Wallis test depending on whether the samples followed a normal distribution or not, respectively. The Mann-Whitney test was used to further analyze amplitudes, with the purpose of comparing stimulation rates and check for statistically significant differences. Statistical significance was attributed to events with *p* ≤ 0.05; a beta error of 0.1 was admitted.

## RESULTS

The sample included 18 subjects (36 ears), 12 females (24 ears) and six males (12 ears). The individuals were aged between 21 and 27 years, and had a mean age of 23.03 ± 1.33 years.

VEMPs were recorded through stimulation and unilateral data acquisition. Proper morphology was attained in 100% of the ears using a stimulation rate of 5.1 stimuli per second; in 96.87% at 10.2 stimuli per second; in 86.11% at 20.4 stimuli per second; and in 72.22% at 40.8 stimuli per second. Tone bursts at 500 Hz were used in acoustic stimulation.

The Kolmogorov-Smirnov revealed latencies and amplitudes followed a normal distribution pattern, except for the latencies seen in wave p13 for stimulation rates of 10.2 and 40.8 stimuli per second. Non-parametric tests were thus used.

The waves were delimited in the test tracings and absolute latencies and amplitudes were determined for waves p13 and n23. Table 1 shows the data related to these parameters for each stimulation rate irrespective of ear.

**Table 1:** VEMP latencies and amplitudes for each stimulation rate.

		Latency (ms)		Amplitude (µV)	
		p13	n23	p13	n23
5.1 stimuli per second	Mean	14.10	24.80	260.43	-328.72
	SD	1.99	2.43	5.68	8.22

10.2 stimuli per second	Mean	14.11	24.65	77.03	-103.11
	SD	2.15	2.52	3.53	1.80

20.4 stimuli per second	Mean	14.20	24.20	60.12	-86.93
	SD	2.74	3.18	2.43	3.71

40.8 stimuli per second	Mean	14.84	24.11	27.92	-23.42
	SD	2.71	4.12	2.15	2.57

*p*-value		0.19**	0.54*	0.06*	0.14*

* ANOVA Test

** Kruskal-Wallis Test.

Table 1 shows that the p13 and n23 wave latencies were kept constant and amplitudes decreased gradually as stimulation rates were increased. However, ANOVA and the Kruskal-Wallis test failed to reveal statistically significant differences between parameters.

Amplitudes were further analyzed by comparing stimulation rates. The Mann-Whitney test revealed statistically significant differences for wave p13 between stimulation rates of 5.1 and 20.4 stimuli per second (*p* = 0.03), and 5.1 and 40.8 stimuli per second (*p* = 0.01). For wave n23, amplitudes were statistically different only when stimulation rates of 5.1 and 40.8 stimuli per second were compared (*p* = 0.02), with no significant differences seen between stimulation rates of 5.1 and 20.4 stimuli per second (*p* = 0.06).

**Graph 1:**
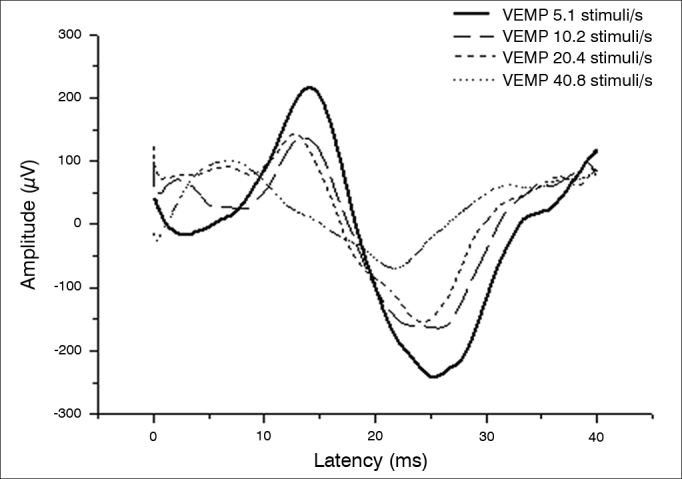
Comparison between amplitudes according to stimulation rate.

Graph 1 shows a comparison between amplitudes at different stimulation rates.

## DISCUSSION

The results seen in the studied population showed that it is possible to consistently acquire records for waves p13 and n23 in the time domain on all analyzed stimulation rates, as similarly reported by other authors^15^, ^26^.

The VEMP latency and amplitude results at a stimulation rate of 5.1 stimuli per second seen in this study were similar to the values reported in the literature^6^, ^27^. As for other stimulation rates, reports in the literature indicate that latency and amplitude tend to decrease as the stimulation rate is increased - a finding in disagreement with this study when latency is considered^15^, ^26^.

Some authors have related the decrease in amplitude consequent to stimulation rate increases to possible SCM muscle fatigue during testing, as subjects are required to produce effective muscle contraction in order to allow for proper recording of potentials. Thus, the longer the tests, the lower the amplitude^27^. Others have described the reduction in amplitude as a consequence of reflex habituation, as the high rate of stimulation exhausts sensory cells and delays the activation of the first neuron^28^, ^29^, ^30^. However, in order to prevent tested individuals from getting tired and to avoid SCM muscle fatigue, the subjects included in this study were asked to rest for one minute between each data acquisition cycle, i.e., when stimulation rates were changed, to prevent muscle fatigue and habituation from occurring.

No statistically significant differences were seen in latency when stimulation rates of 5.1 and 10.2 stimuli per second were compared, as also reported by other authors^15^, ^26^. However, most choose a rate of five stimuli per second, as it allows for more consistent data acquisition and easier identification of tracings^26^.

Lastly, the rate of 10.2 stimuli per second appears to be more adequate in clinical applications as it produces waves with more adequate morphology, with equal tracing identification and amplitudes which are not statistically different when compared to the rate of five stimuli per second, in addition to being visible to the naked eye. Additionally, tests performed at a rate of 10.2 stimuli per second reduce patient discomfort and data acquisition times^15^. Further studies with larger populations are yet required to confirm the adjustments recommended for the testing protocol.

## CONCLUSION

The highest reliable stimulation rate observed for the population included in this study was 10.2 stimuli per second.

## References

[1] Damen MMJ (2007). Clinical application of the threshold. Medical Engineering.

[2] Pollak L, Kushnir M, Stryjer R (2006). Diagnostic value of vestibular evoked myogenic potentials in cerebellar and lower-brainstem strokes. Neurophysiol Clin.

[3] Rauch SD (2006). Vestibular evoked myogenic potentials. Curr Opin Otolaryngol Head Neck Surg.

[4] Sazgar AA, Dortaj V, Akrami K, Akrami S, Karimi Yazdi AR (2006). Saccular damage in patients with high-frequency sensorineural hearing loss. Eur Arch Otorhinolaryngol.

[5] Lütkenhöner B, Stoll W, Basel T (2010). Modeling the vestibular evoked myogenic potential. J Theor Biol.

[6] Akin FW, Murnane OD, Panus PC, Caruthers SK, Wilkinson AE, Proffitt TM (2004). The influence of voluntary tonic EMG level on the vestibular-evoked myogenic potential. J Rehabil Res Dev.

[7] Halmagyi GM, Colebatch JG, Curthoys IS (1994). New tests of vestibular function. Baillieres Clin Neurol.

[8] Hong SM, Park DC, Yeo SG, Cha CI (2008). Vestibular evoked myogenic potentials in patients with benign paroxysmal positional vertigo involving each semicircular canal. Am J Otolaryngol.

[9] Pérez Guillén V, González García E, García Piñero A, Piqueras Del Rey A, Morera Pérez C, Pérez Garrigues H. (2005). Vestibular evoked myogenic potential: a contribution to the vestibular physiology and pathology knowledge. Quantitative patterns in healthy subjects. Acta Otorrinolaringol Esp.

[10] Shimizu K, Murofushi T, Sakurai M, Halmagyi M (2000). Vestibular evoked myogenic potentials in multiple sclerosis. J Neurol Neurosurg Psychiatry.

[11] Colebatch JG, Halmagyi GM (1992). Vestibular evoked potentials in human neck muscles before and after unilateral vestibular deafferentation. Neurology.

[12] Burkard RF, Eggermont JJ, Don M (2007). Auditory evoked potentials: Basic principles and Clinical Application.

[13] Timmer FC, Zhou G, Guinan JJ, Kujawa SG, Herrmann BS, Rauch SD (2006). Vestibular evoked myogenic potential (VEMP) in patients with Ménière's disease with drop attacks. Laryngoscope.

[14] Basta D, Todt I, Ernst A (2005). Normative data for P1/N1-latencies of vestibular evoked myogenic potentials induced by air- or bone-conducted tone bursts. Clin Neurophysiol.

[15] Sheykholeslami K, Habiby Kermany M, Kaga K (2001). Frequency sensitivity range of the saccule to bone-conducted stimuli measured by vestibular evoked myogenic potentials. Hear Res.

[16] Kelsch TA, Schaefer LA, Esquivel CR (2006). Vestibular evoked myogenic potentials in young children: test parameters and normative data. Laryngoscope.

[17] Huang TW, Su HC, Cheng PW (2005). Effect of click duration on vestibular-evoked myogenic potentials. Acta Otolaryngol.

[18] Cheng PW, Murofushi T (2001). The effects of plateau time on vestibular-evoked myogenic potentials triggered by tone bursts. Acta Otolaryngol.

[19] Cheng PW, Murofushi T (2001). The effect of rise/fall time on vestibular-evoked myogenic potential triggered by short tone bursts. Acta Otolaryngol.

[20] Murofushi T, Matsuzaki M, Wu CH (1999). Short tone burst-evoked myogenic potentials on the sternocleidomastoid muscle: are these potentials also of vestibular origin?. Arch Otolaryngol Head Neck Surg.

[21] Stapells DR, Seewald RC, Bamford J. (2005). A sound foundation through early amplification.

[22] Picton TW, John MS, Dimitrijevic A, Purcell D (2003). Human auditory steady-state responses. Int J Audiol.

[23] Felipe L, Kigman H (2012). Gonçalves DH. Potencial evocado miogênico vestibular. Arq Int Otorrinolaringol.

[24] Lins OG (2002). Audiometria fisiológica tonal utilizando respostas de estado estável auditivas do tronco cerebral [Tese de Doutorado].

[25] Pauli-Magnus D, Hoch G, Strenzke N, Anderson S, Jentsch TJ, Moser T (2007). Detection and differentiation of sensorineural hearing loss in mice using auditory steady-state responses and transient auditory brainstem responses. Neuroscience.

[26] Wu CH, Murofushi T (1999). The effect of click repetition rate on vestibular evoked myogenic potential. Acta Otolaryngol.

[27] Oliveira AC (2010). Estudo dos potenciais evocados miogênicos vestibulares de estado estável [Tese de doutorado].

[28] Murofushi T, Curthoys IS (1997). Physiological and anatomical study of click-sensitive primary vestibular afferents in the guinea pig. Acta Otolaryngol.

[29] Murofushi T, Curthoys IS, Topple AN, Colebatch JG, Halmagyi GM (1995). Responses of guinea pig primary vestibular neurons to clicks. Exp Brain Res.

[30] Murofushi T, Curthoys IS, Gilchrist DP (1996). Response of guinea pig vestibular nucleus neurons to clicks. Exp Brain Res.

